# Gnotobiotic IL-10^−/−^; NF-κB^EGFP^ Mice Develop Rapid and Severe Colitis Following *Campylobacter jejuni* Infection

**DOI:** 10.1371/journal.pone.0007413

**Published:** 2009-10-20

**Authors:** Elisabeth Lippert, Thomas Karrasch, Xiaolun Sun, Brigitte Allard, Hans H. Herfarth, Deborah Threadgill, Christian Jobin

**Affiliations:** 1 Department of Medicine and Center for Gastrointestinal Biology and Disease, University of North Carolina at Chapel Hill, Chapel Hill, North Carolina, United States of America; 2 Department of Internal Medicine I, University of Regensburg, Regenburg, Germany; 3 Department of Genetics, University of North Carolina at Chapel Hill, Chapel Hill, North Carolina, United States of America; 4 Department of Pharmacology, University of North Carolina at Chapel Hill, Chapel Hill, North Carolina, United States of America; 5 Department of Microbiology, North Carolina State University, Raleigh, North Carolina, United States of America; Charité-Universitätsmedizin Berlin, Germany

## Abstract

Limited information is available on the molecular mechanisms associated with *Campylobacter jejuni* (*C. jejuni*) induced food-borne diarrheal illnesses. In this study, we investigated the function of TLR/NF-κB signaling in *C. jejuni* induced pathogenesis using gnotobiotic IL-10^−/−^; NF-κB^EGFP^ mice. In vitro analysis showed that *C. jejuni* induced IκB phosphorylation, followed by enhanced NF-κB transcriptional activity and increased IL-6, MIP-2α and NOD2 mRNA accumulation in infected-mouse colonic epithelial cells CMT93. Importantly, these events were blocked by molecular delivery of an IκB inhibitor (Ad5IκBAA). NF-κB signalling was also important for *C.jejuni*-induced cytokine gene expression in bone marrow-derived dendritic cells. Importantly, *C. jejuni* associated IL-10^−/−^; NF-κB^EGFP^ mice developed mild (day 5) and severe (day 14) ulcerating colonic inflammation and bloody diarrhea as assessed by colonoscopy and histological analysis. Macroscopic analysis showed elevated EGFP expression indicating NF-κB activation throughout the colon of *C. jejuni* associated IL-10^−/−^; NF-κB^EGFP^ mice, while fluorescence microscopy revealed EGFP positive cells to be exclusively located in lamina propria mononuclear cells. Pharmacological NF-κB inhibition using Bay 11-7085 did not ameliorate *C. jejuni* induced colonic inflammation. Our findings indicate that *C. jejuni* induces rapid and severe intestinal inflammation in a susceptible host that correlates with enhanced NF-κB activity from lamina propria immune cells.

## Introduction

The gram-negative *Campylobacter jejuni* (*C. jejuni*) is the most prevalent cause of bacterial-food borne diarrheal diseases worldwide with up to 2.4 million cases annually in the United States alone [1]. This microorganism colonizes the cloaca, cecum and large intestine of birds and the ileum and colon of humans. The main sources of transmission to humans are the consumption and handling of contaminated poultry, and to a lesser extent ingestion of contaminated water [2]–[5]. Although poultry which are frequently colonized with up to 10^10^ *C. jejuni* bacteria/g of cecal content remain healthy, infected humans develop gastroenteritis with as few as 100–500 *C.jejuni* (strain 81–176) [6]. Clinical features of *C. jejuni* infection range from severe inflammatory bloody diarrhea to mild non-inflammatory watery diarrhea accompanied by acute abdominal pain and fever, which lasts between 24–48 h and generally resolves after 10 days [5]. Although the infection is self limiting in healthy persons, reactive arthritis, gastrointestinal (reactivation of inflammatory bowel diseases) and neurological (Guillian-Barré-Syndrome) disorders have been reported in susceptible hosts [3]. *C. jejuni* is a microaerophilic, motile Gram negative invasive bacterium from the family Campylobacteraceae that possesses a single polar flagellum at one or both ends [7]. Although the rate of *C. jejuni* infection exceeds that of salmonellosis and shigellosis, a paucity of information exists on the pathogenesis and host response to this microorganism [8]–[11]. This is likely due to the lack of reliable experimental murine models reproducing various aspects of *C. jejuni* pathogenesis [11]. Murine models of *C.jejuni* infection are typically viewed as colonization rather than disease models, thereby limiting the generation of new knowledge regarding host responses to this pathogen [11]–[14].

Recent reports utilizing new genetically deficient mice have shed light on *C. jejuni*-induced pathogenesis. For example, Muc-1^−/−^ mice are more susceptible to *C. jejuni* colonization and displayed small intestinal epithelial damage [15]. This indicates that production of the mucin layer represents an important host innate protective mechanism against *C. jejuni*-mediated pathogenesis. Subsequent reports identified the antimicrobial Nramp1 protein as an important regulator of *C. jejuni*-induced pathogenesis [13], [16], [17]. Mice deficient for both the TLR signaling protein MyD88 and the antimicrobial protein Nramp1 were more susceptible to *C. jejuni* colonization when administered systemically. Although useful, these new findings provide limited insight into the inflammatory host response to *C. jejuni* infection and ensuing diarrhea/inflammation. However, two recent reports have provided a potential new model to study *C. jejuni*-induced colitis. Using IL-10^−/−^ mice housed in specific pathogen free (SPF) conditions, Mansfield and colleagues demonstrated that enteritis developed in ∼50–80% of *C. jejuni* (NCTC 11168) colonized IL-10^−/−^ mice after more than 28 days of infection depending on the genetic background [18], [19]. Fairly high doses of inoculum (10^6^–10^10^ colony forming units (cfu)) were necessary to achieve colonization and inflammatory response in this model. Nevertheless, these findings indicate that *C. jejuni* colonizes the intestinal tract of murine hosts exhibiting defective innate or immunoregulatory responses. Despite the important progress made using these new models, the molecular mechanism responsible for *C. jejuni*-mediated pathogenesis remains largely elusive. In this study, we utilize an in vitro and in vivo approach to dissect the function of TLR/NF-κB signaling in *C. jejuni*-induced pathogenesis.

## Materials and Methods

### Ethics statement

All animal protocols were approved by the Institutional Animal Care and Use Committee of the University of North Carolina at Chapel Hill.

### Mice

Germ-free IL-10^wt/wt^; NF~κB^EGFP^, IL-10^−/−^; NF-κB^EGFP^ (129/SvEv;C57/Bl/6 mixed background) mice [20] and IL-10^−/−^ and WT (129/SvEv) mice were associated with various doses of *C. jejuni* (Strain 81–176; 10^2^, 10^4^, 10^6^, and 10^8^ cfu/mouse). Mice were sacrificed at 14 days. Mice were housed under germ-free conditions at the Gnotobiotic Animal Facility at the University of North Carolina at Chapel Hill and associated with 10^8^ cfu/mouse. Mice were sacrificed after 14 days. Mice were monitored weekly for *C. jejuni* association with PCR or stool culture using the following primer pair: TGTTGAAGGGTTTGAAGAGC (forward), GCAAGTTGACCCTCTTCTATGG (reverse). In addition, *C. jejuni* association and absence of contamination by other bacterial species were confirmed by periodic culture of stool samples. For inhibition of NF-κB in vivo, mice were treated with Bay 11–7085 (Calbiochem, La Jolla, CA) i.p. (200 µg/kg) one day before *C. jejuni* association and then injected three times per week. After completion of the experiment, mice were euthanized with CO_2_ intoxication. Proximal and distal colon, cecum, small intestine, mesenteric lymph nodes (MLN), spleen and liver were collected from each mouse and used for further processing of RNA, protein and tissue cultures on regular agar plates (Mueller Hinton Agar, Remel, Lenexa, KS) or on Brucella Agar plates (Remel) over 24–48 h. Proximal and distal colon as well as cecum and small intestine were fixed in 10% buffered formalin (Fisher Scientific, Pittsburgh, PA, USA) overnight, paraffin-embedded, sectioned, and stained with H&E for histological evaluation. Histological assessment was performed by two blinded investigators using a score as described previously [21]. Briefly, mucosal inflammation was scored by evaluation of the degree of lamina propria mononuclear cell infiltration, loss of goblet cells, architectural distortion as well as crypt hyperplasia using a score from 0–4.

### *C. jejuni* preparation

*C. jejuni* strain 81–176 (human isolate) was grown under microaerophilic conditions on regular agar plates over 24–48 hours using a MACS VA 500 microaerophilic workstation (Don Whitley Scientific, Microbiology International, Frederick, MD). The bacteria were resuspended in Tryptic Soy Broth (Teknova, Hollister, CA), counted and diluted to a final concentration ranging between 10^2^, 10^4^, 10^6^, 10^8^ and 10^9^ cfu/ml. Each culture was gram stained (Fisher Scientific) and checked for pure culture conditions under the microscope for each experiment.

### Cell lines

The immortalized murine rectum carcinoma cell line CMT-93 was cultured as described previously [22]. Cells were incubated with *C.jejuni* (moi 50) in 1% medium for different time points. Supernatant and cells were collected for further analysis in 1X Laemmli-buffer for protein analysis and Trizol (Invitrogen, Carlsbad, CA) for RNA analysis.

### RNA extraction and amplification by RT-PCR

RNA was isolated using TRIzol (Invitrogen), reverse transcribed and amplified as previously described using primers specific for murine MIP-2, IL-12p40, TNFα, IL-6, and GAPDH [20], [23]. For PCR analysis, products were subjected to electrophoresis on 2% agarose gels containing GelStar fluorescent dye (Cambrex BioScience Rockland). Fluorescence staining was captured using an Alpha Imager 2000 (Alpha Innotech, San Leandro, CA). Cytokine expression was quantified using real-time PCR (Applied Biosystems 7900HT Fast Real-Time PCR System). Primer sequences were as follows;

**Table:** 

GATCTTCATGAGGTAGTCTGT	β-actin reverse
CCAACCGTGAAAAGATGACC	β-actin forward
GGTGAAGGTCGGTGTGAACGGA	GAPDH forward
GTGGGGTCTCGCTCCTGGAAGA	GAPDH reverse
CGGAGGCTTGGTTACACATGTT	IL-6 forward
CTGGCTTTGTCTTTCTTGTTATC	IL-6 reverse
TACAGGCTTGTCACTCGAATT	TNFα forward
ATGAGCACAGAAAGCATGATG	TNFα reverse
CACGGCAGCAGAATAAATATG	IL-12p40 forward
TTGCATTGGACTTCGGTAGA	IL-12p40 reverse
CCGCTGTTGTGGCCAGTGAACTGCG	MIP-2 forward
TTAGCCTTGCCTTTGTTCAGTAT	MIP-2 reverse

### Western blot

Proteins were separated using SDS-PAGE and transferred to nitrocellulose membranes. Antibodies to IκBα and RelA were diluted 1∶1000 in 0.1% TBS-Tween with 5% dry milk. Immunoreactive proteins were detected using the enhanced chemiluminescence light (ECL) detecting kit (Amersham Biosciences, Piscataway, NJ) as described previously [23].

### Adenoviruses and cell infection

CMT-93 cells were infected with the adenoviral vector Ad5IκBAA in serum-reduced media (1%) at optimal multiplicity of infection (MOI) for 12 hr. The adenovirus was washed off, fresh serum-reduced medium was added and cells were stimulated with *C. jejuni* (moi 50) and LPS (5 µg/ml). Cells were pretreated with cyclohexamide (CHX, 50 µg/ml Sigma) to prevent IκB resynthesis.

### Assessment of enhanced EGFP expression

NF-κB^EGFP^, IL-10^wt/wt^; NF-κB^EGFP^ and IL-10^−/−^; NF-κB^EGFP^ mice were sacrificed at the indicated times and the entire colon dissected and then directly imaged for EGFP expression as described previously [20]. For tissue sections, colons were resected from mice following infection as described in the text, fixed in 4% paraformaldehyde (PFA) for 10–24 hours, washed twice in PBS, and transferred to vials containing 30% sucrose for 24 hours. Five to seven micron (µm) sections were cut on a cryostat and counter-stained with DAPI. EGFP expression was imaged using an Olympus IX70 (Olympus, Melville, NY) fitted with EGFP-specific filters (Omega Optical, XF116-2, Brattleboro, VT). Images were captured using a digital SPOT^TM^ camera (Diagnostic Instruments Inc., McHenry, IL). Identical exposure times were used for each data point within an individual experiment.

### Colonoscopy

Direct visualization of the colon in vivo was performed using a “Coloview system” (Karl Storz Veterinary Endoscopy). Mice were supplied with food and water until the endoscopy was performed. If fecal material obstructed the view of the endoscope, colons were flushed with 0.9% saline. For the colonoscopies, mice were anesthetized with 1.5 to 2% isoflurane and 3 to 4 cm of the colon from the anal verge until the splenic flexure was visualized after inflation of the colon with air. The colonoscopic procedures were digitally recorded on an AIDA Compaq PC.

### Statistical analysis

Data are expressed as mean +/− standard deviation. Statistical analysis was performed using the Mann-Whitney test (histology) and students t-test (remaining data) and using SigmaPlot for Windows Version 8.0 and SigmaStat for Windows Version 3.5. The t-test was used to investigate whether the means of two groups are statistically different from each other. This analysis is appropriate whenever one wants to compare the means of two groups. The Mann-Whitney test was used as a non-parametric test for assessing whether two independent samples of observations came from the same distribution. Differences were considered significant with a p-value of <0.05.

## Results

### *C. jejuni* induces NF-κB-signaling and gene expression in intestinal epithelial cells and in bone marrow-derived dendritic cells (BMDC)

**Figure 1:**
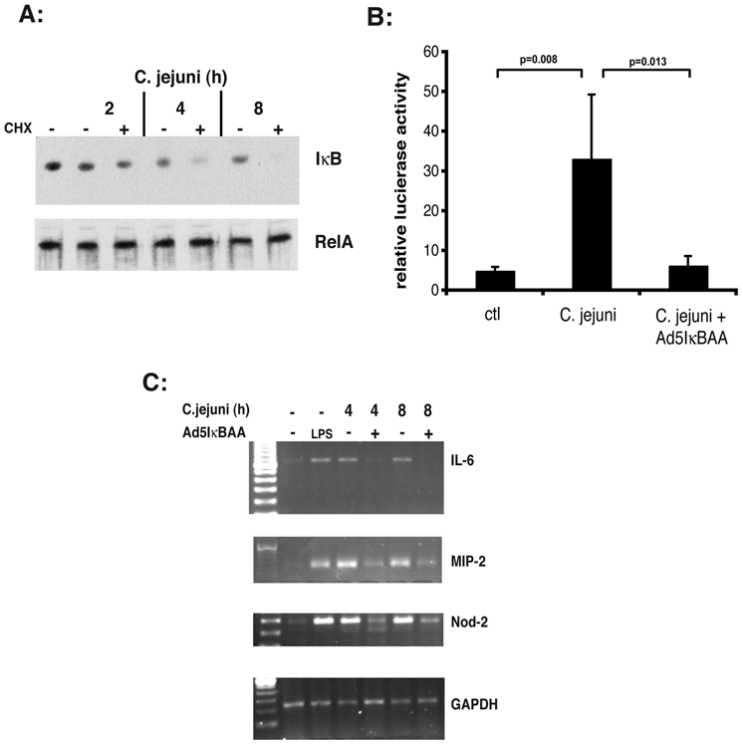
*C. jejuni* induces NF-κB-signalling and gene expression in CMT-93 cells. (A) *C. jejuni* infection induced IκB degradation in CMT93 cells. Cells were infected with *C. jejuni* (MOI 50) for the indicated time points in presence or absence of cycloheximide (CHX; 25 µg/ml). Total protein was extracted and 20 µg was subjected to SDS-PAGE followed by immunoblotting with IκBα and RelA specific antibodies. (B) *C. jejuni* infection induced NF-κB transcriptional activity in CMT93 cells. CMT-93 cells were infected with the reporter Ad5NF-κB-LUC adenoviral vector and where indicated co-infected with Ad5IκBAA (MOI: 50) to block the κB-luciferase activity. Cells were then infected with *C. jejuni* (MOI 50) for 24 h and luciferase activity measured after 16 h using an Lmax microplate reader. Results were normalized for extract protein concentrations (*p = 0.008 control vs. *C. jejuni* infected cells; p = 0.013 *C. jejuni* vs. *C. jejuni*-Ad5IκBAA). (C) Increased IL-6, MIP-2 and NOD2 mRNA accumulation in *C. jejuni* infected CMT93 cells. CMT-93 cells were infected with Ad5IκBAA and then stimulated with LPS (5 µg/ml) or infected with *C. jejuni* (MOI 50) for the indicated time points. Cells were lysed in trizol, and total RNA was extracted, reverse-transcribed (1 µg), and amplified by PCR using specific murine IL-6, MIP-2 and NOD2 primers. The housekeeping gene GAPDH was utilized to ascertain similar loading. Results are representative of 3 independent experiments.

We first investigated the impact of NF-κB signaling on *C. jejuni* mediated host response using the murine colonic cell line CMT-93. As seen in Fig. 1A, *C. jejuni* (MOI 10) time-dependently induced IκBα degradation as measured by Western blot analysis, a process better appreciated when cells were exposed to the protein synthesis inhibitor cyclohexamide (CHX). In addition, NF-κB transcriptional activity significantly increased (>6 fold) in *C. jejuni*-infected CMT93 cells (Fig. 1B). Importantly, the IκB superrepressor delivered by adenoviral vector (Ad5IκBAA) significantly reduced *C. jejuni*-induced NF-κB transcriptional activity in CMT-93 cells (Fig. 1B). To investigate the impact of NF-κB signaling on *C. jejuni*-induced endogenous gene expression, we next measured IL-6, MIP-2 and NOD2 mRNA accumulation in cells expressing the IκB super-repressor. Interestingly, *C. jejuni*–induced IL-6 and MIP-2 mRNA accumulation was strongly blocked by Ad5IκBAA (Fig. 1C).

**Figure 2:**
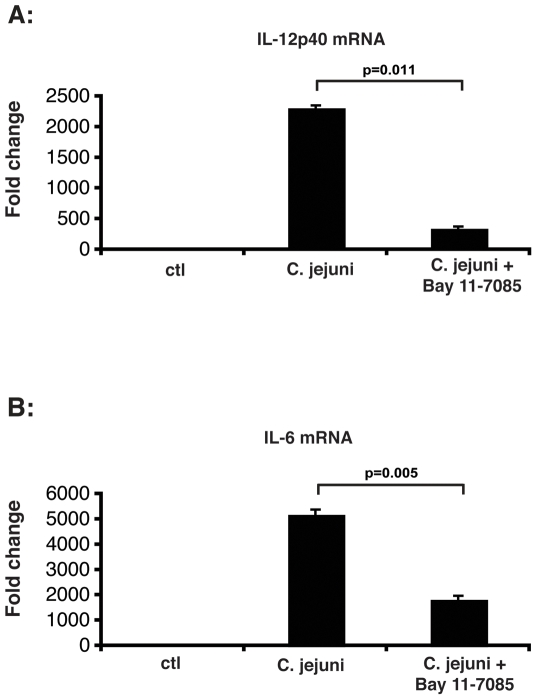
NF-κB signaling drives *C. jejuni* induced cytokine gene expression in BMDC. BMDC were generated from WT mice and treated with Bay 11-7085 (10 µM) and then stimulated with *C. jejuni* (MOI 50) for 4 h. Cells were lysed in Trizol, total RNA was extracted, reverse transcribed and IL-12p40 (A) and IL-6 (B) mRNA expression was detected using real-time PCR (Applied Biosystems 7700 sequence detection system). Results were normalized to the housekeeping gene GAPDH to ascertain similar loading. Results are the mean +/− SD of triplicates samples and are from one of three independent experiments (*p = 0.011 for IL-12p40 vs. Bay 11-7085-treated cells; *p = 0.005 for IL-6 vs. Bay 11-7085-treated cells).

Activation of TLR signalling by antigen presenting cells such as dendritic cells plays a key role in the innate host response to invasive pathogenic bacteria. We next investigated the role of NF-κB signalling in *C.jejuni* induced host response in BMDC generated from WT mice using the pharmacological inhibitor Bay 11-7085. Fig. 2 showed that *C.jejuni*-induced IL-12p40 (Fig. 2A) and IL-6 (Fig. 2B) mRNA accumulation was strongly reduced in Bay 11-7085-treated BMDC compared to untreated cells. In summary, *C. jejuni* infection induces the classical NF-κB activation pathway in vitro, leading to increased expression of NF-κB dependent genes in mouse colonic epithelial CMT-93 cells and in BMDC.

### *C. jejuni-mono* associated IL-10^−/−^; NF-κB^EGFP^ mice display rapid, severe and progressive colonic inflammation

**Figure 3:**
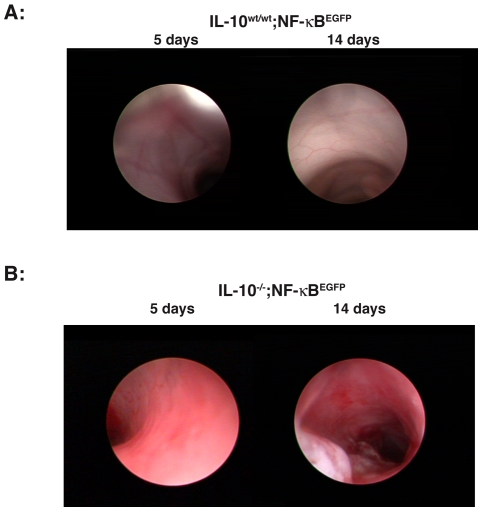
*C. jejuni* induces rapid and progressive severe colonic inflammation in IL-10^−/−^; NF-κB^EGFP^ mice. Gnotobiotic IL-10^−/−^; NF-κB^EGFP^ mice and control IL-10^wt/wt^; NF-κB^EGFP^ mice were associated with *C. jejuni* by oral gavage (10^8^ cfu/mouse). Inflammation was evaluated macroscopically in vivo using a murine miniature endoscope in IL-10^wt/wt^; NF-κB^EGFP^ control mice (Fig. 2A, n = 13) and IL-10^−/−^; NF-κB^EGFP^ mice on day 5 and 14 (Fig. 2B, n = 12). The data are representative for two additional experiments.

**Figure 4:**
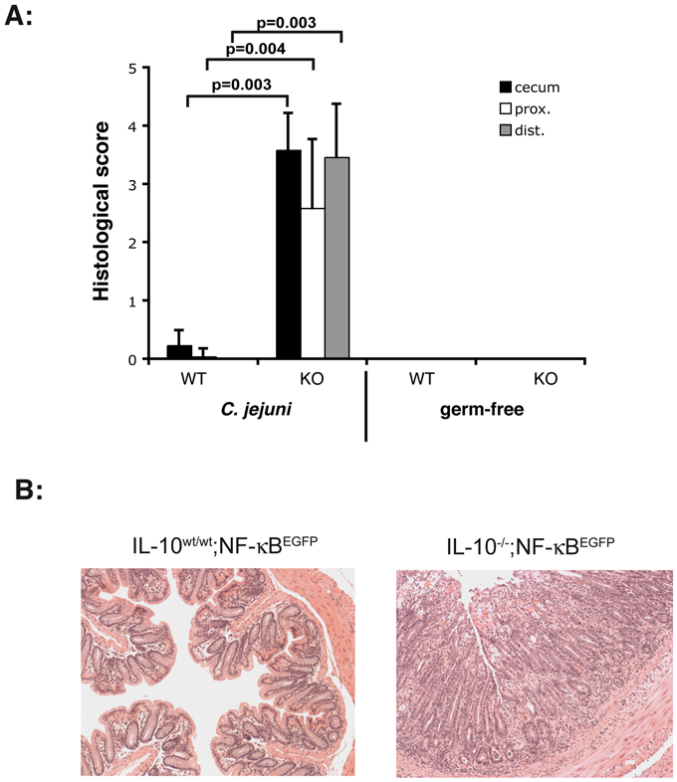
Histology showed strong signs of inflammation in IL-10^−/−^; NF-κB^EGFP^ mice. Gnotobiotic IL-10^−/−^; NF-κB^EGFP^ mice were associated with *C. jejuni* by oral gavage (10^8^ cfu/mouse) for 14 days. Mice were euthanized and different parts of the colon were paraffin-embedded and used for H&E staining. Histological analysis of *C. jejuni*-associated IL-10^−/−^; NF-κB^EGFP^ and IL-10^wt/wt^; NF-κB^EGFP^ was performed as described in Materials and Methods. Histological score revealed statistical significant difference between IL-10^wt/wt^; NF-κB^EGFP^ and IL-10^−/−^; NF-κB^EGFP^ associated with *C. jejuni* (cecum *p = 0.003, prox *p = 0.004, dist *p = 0.003). IL-10^wt/wt^; NF-κB^EGFP^: n = 8, IL-10^−/−^; NF-κB^EGFP^: n = 4, IL-10^wt/wt^; NF-κB^EGFP^ *C. jejuni* associated: n = 13, IL-10^−/−^; NF-κB^EGFP^ *C. jejuni* associated: n = 12. WT = IL-10^wt/wt^; NF-κB^EGFP^, KO = IL-10^−/−^; NF-κB^EGFP^.

We previously reported that gnotobiotic IL-10^−/−^ mice represent a powerful tool to study host response to commensal bacteria colonization [20]. Germ-free IL-10^−/−^; NF-κB^EGFP^ mice and control IL-10^wt/wt^; NF-κB^EGFP^ mice were associated with *C. jejuni* by oral gavage (10^8^ cfu/mouse) and inflammation was evaluated macroscopically after 5 and 14 days using a murine miniature endoscope. Interestingly, *C. jejuni* induces rapid and progressive severe, ulcerating colonic inflammation with bloody diarrhea in IL-10^−/−^; NF-κB^EGFP^ mice (Fig. 3B), whereas IL-10^wt/wt^; NF-κB^EGFP^ control mice demonstrate macroscopically healthy colonic mucosa (Fig. 3A). To provide a more accurate assessment of *C. jejuni* induced colonic inflammation, colons of 14- day *C. jejuni* associated IL-10^−/−^; NF-κB^EGFP^ and IL-10^wt/wt^; NF-κB^EGFP^ mice were dissected, fixed and sectioned for histological analysis. As expected, *C. jejuni*-associated IL-10^−/−^; NF-κB^EGFP^ mice showed strong signs of inflammation in the cecum, proximal and distal colon whereas *C. jejuni* associated IL-10^wt/wt^; NF-κB^EGFP^ mice displayed minimal inflammation (Fig. 4A). Representative histological sections from *C. jejuni*-associated IL-10^−/−^; NF-κB^EGFP^ mice showed clear signs of inflammation after 14 days with crypt hyperplasia, goblet cell depletion, immune cell infiltration and ulcers. IL-10^wt/wt^; NF-κB^EGFP^ mice displayed normal physiologic cell structure (Fig. 4B). Polymerase chain reaction (PCR) as well as serial bacterial cultures performed in sterilely harvested stool samples confirmed the presence of viable *C. jejuni* in both IL-10^wt/wt^; NF-κB^EGFP^ and IL-10^−/−^; NF-κB^EGFP^ mice 14 days after association, indicating that both gnotobiotic IL-10^wt/wt^; NF-κB^EGFP^ and IL-10^−/−^; NF-κB^EGFP^ mice are colonized by this microorganism (data not shown).

**Figure 5:**
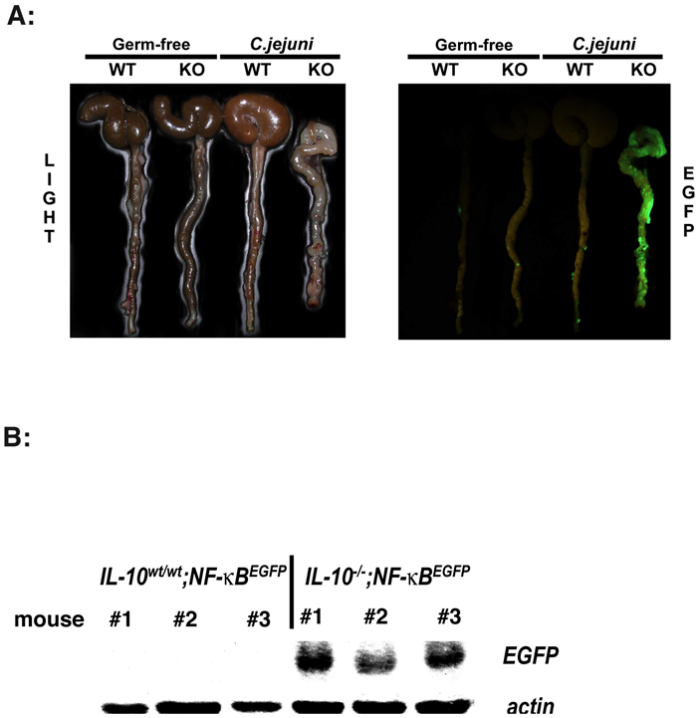
*C. jejuni* induces NF-κB signaling in IL-10^−/−^; NF-κB^EGFP^ mice. (A) *C. jejuni* induces NF-κB activity in the colon of IL-10^−/−^; NF-κB^EGFP^ mice. IL-10^wt/wt^; NF-κB^EGFP^ and IL-10^−/−^; NF-κB^EGFP^ mice were colonized for 14 days with *C. jejuni* (10^8^ cfu/mouse), mice were euthanized, colons dissected and EGFP expression immediately visualized using a CCD camera in a light-tight imaging box with a dual filtered light source. The data are representative of three independent experiments. IL-10^wt/wt^; NF-κB^EGFP^: n = 8, IL-10^−/−^; NF-κB^EGFP^: n = 4, IL-10^wt/wt^; NF-κB^EGFP^ *C. jejuni* associated: n = 13, IL-10^−/−^; NF-κB^EGFP^ *C. jejuni* associated: n = 12. (B) *C. jejuni* induces elevated EGFP protein expression in the colon. Colonic protein of *C. jejuni* associated IL-10^wt/wt^; NF-κB^EGFP^and IL-10^−/−^; NF-κB^EGFP^ were extracted and 20 µg were used for SDS-PAGE gel analysis. Gels were stained with EGFP specific antibodies. n = 3 per group.

**Figure 6:**
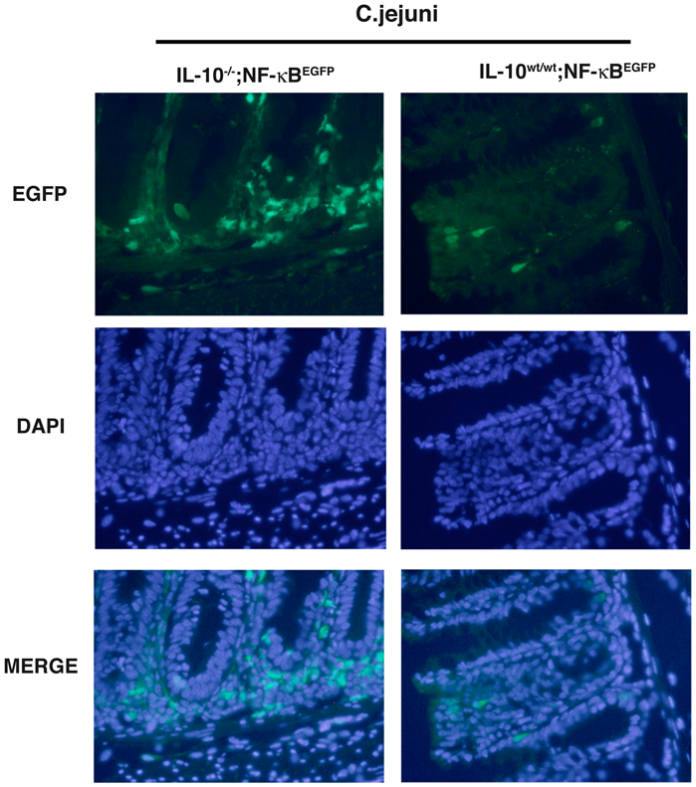
EGFP expression is predominantly found in lamina propria mononuclear cells of *C. jejuni*-infected IL-10^−/−^; NF-κB^EGFP^ mice. Colonic sections from 14 days *C. jejuni* associated IL-10^wt/wt^; NF-κB^EGFP^ and IL-10^−/−^; NF-κB^EGFP^ mice were examined by fluorescent microscopy. The localization of EGFP positive cells was determined by simultaneous EGFP and DAPI analysis.

**Figure 7:**
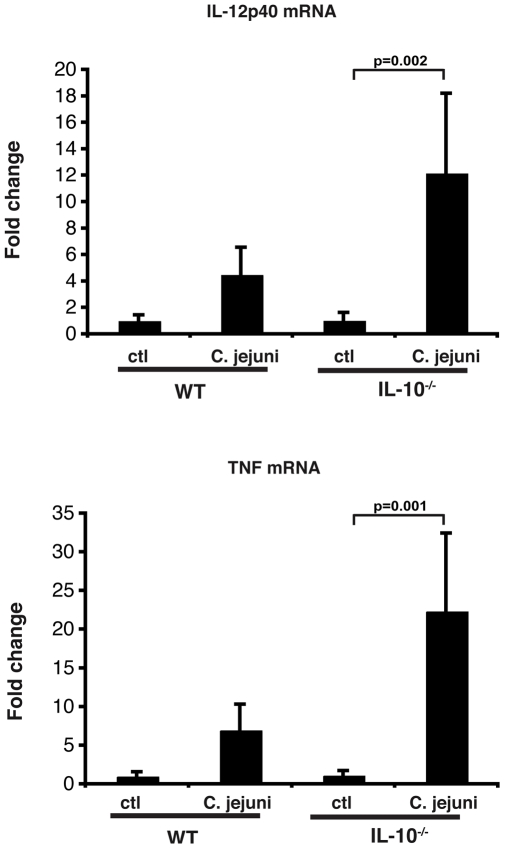
Increased TNFα and IL-12p40 mRNA accumulation in *C. jejuni*-associated IL-10^−/−^; NF-κB^EGFP^ mice. IL-10^wt/wt^; NF-κB^EGFP^ and IL-10^−/−^; NF-κB^EGFP^ were associated with *C. jejuni* for 14 days. Colonic sections were lysed in trizol and RNA was extracted, reverse-transcribed (1 µg) and amplified by PCR using specific murine TNFa and IL12p40 primers. GAPDH was used as loading control. WT = IL-10^wt/wt^; NF-κB^EGFP^, n = 7; KO = IL-10^−/−^; NF-κB^EGFP^, n = 7; WT *C. jejuni* associated: n = 12; KO *C. jejuni* associated: n = 10. (*p = 0.002 for IL12p40 vs. control, *p = 0.001 for TNF vs. control).

To gain more insight into the signalling event associated with *C. jejuni* induced colitis, IL-10^wt/wt^; NF-κB^EGFP^ and IL-10^−/−^; NF-κB^EGFP^ mice were colonized for 14 days and their colons dissected and EGFP expression visualized using a CCD camera in a light-tight imaging box with a dual filtered light source. *C. jejuni* associated IL-10^−/−^; NF-κB^EGFP^ mice exhibited enhanced EGFP expression (NF-κB activity) over the entire length of the colon as compared to IL-10^wt/wt^; NF-κB^EGFP^ mice (Fig. 5A). Western blot analysis of whole colon protein extracts showed elevated EGFP expression in *C. jejuni*-associated IL-10^−/−^; NF-κB^EGFP^ compared to IL-10^wt/wt^; NF-κB^EGFP^ mice (Fig. 5B). To further define cell types responsible for the overall macroscopic increase in EGFP expression, colonic sections from 14-day *C. jejuni* associated IL-10^wt/wt^; NF-κB^EGFP^ and IL-10^−/−^; NF-κB^EGFP^ mice were examined. Of note, *C. jejuni* induced EGFP expression was mostly confined to lamina propria mononuclear cells located in-between the crypts in IL-10^−/−^; NF-κB^EGFP^ mice whereas EGFP expression was minimal in IL-10^wt/wt^; NF-κB^EGFP^ mice (Fig. 6). In accordance with enhanced NF-κB activity (EGFP expression), significantly increased TNF and IL-12p40 mRNA accumulation was detected in *C. jejuni*-associated IL-10^−/−^; NF-κB^EGFP^ mice compared to IL-10^wt/wt^; NF-κB^EGFP^ mice (Fig. 7). Although asymptomatic, increased TNF and IL-12p40 mRNA accumulation was observed in *C. jejuni*-associated IL-10^wt/wt^; NF-κB^EGFP^ mice compared to germ-free mice.

**Figure 8:**
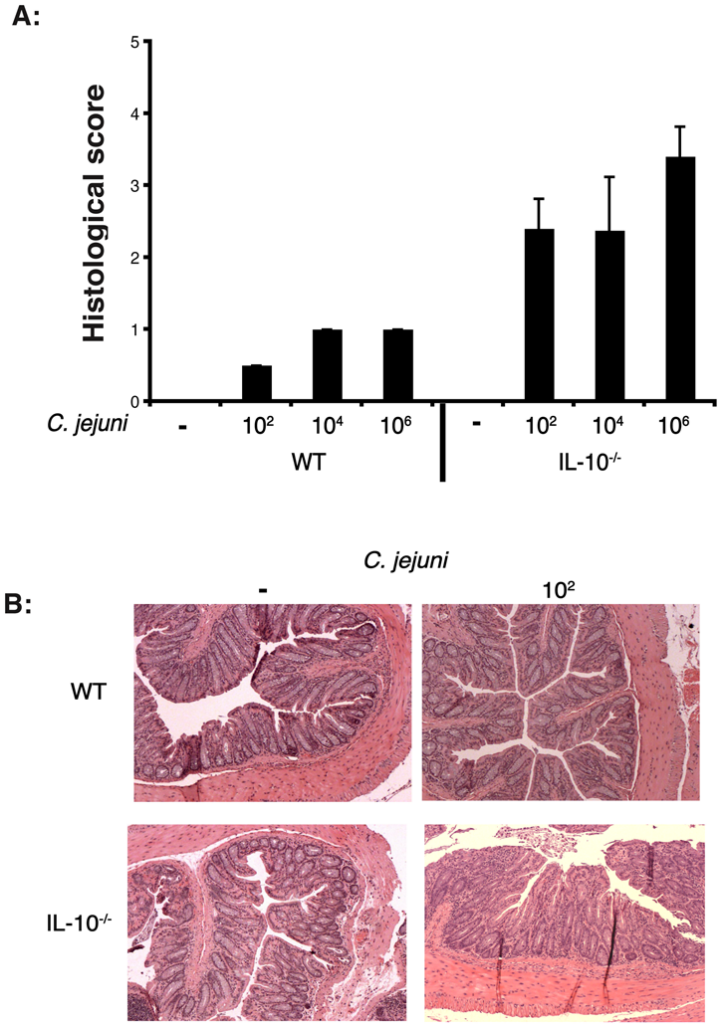
Low *C. jejuni* inoculum induced colitis in IL-10^−/−^ mice. IL-10^−/−^ mice were infected with different amounts of *C. jejuni* (10^2^, 10^4^, and 10^6^ cfu/mouse) for 14 days. (A) The degree of intestinal inflammation was determined by histological analysis of colonic sections as described. (B) Representative section of the colon of IL-10^−/−^ mice infected with 10^2^ bacteria. n = 5 per group, magnification 100x.

We next tested the sensitivity of our infection protocol in gnotobiotic IL-10^−/−^ mice. Germ-free mice were infected with different *C. jejuni* amounts (10^2^, 10^4^, and 10^6^ cfu/mouse) for 14 days and the degree of intestinal inflammation was determined by histological analysis (Fig. 8A). Interestingly, the intestine of IL-10^−/−^ mice infected with as low as 10^2^ bacterium displayed loss of architecture, loss of goblet cells, immune cell infiltration and epithelial hyperplasia in the cecum, proximal and distal colon, indicating that germ-free IL-10^−/−^ mice are exquisitely sensitive to *C. jejuni* infection (Fig. 8B). These findings demonstrate that germ-free IL-10^−/−^ mice develop rapid and severe intestinal inflammation following *C. jejuni* infection, which correlates with activation of NF-κB signalling.

To further define the role of NF-κB signalling in the development of *C.jejuni*-induced colitis, NF-κB activation was pharmacologically inhibited in gnotobiotic IL-10^−/−^; NF-κB^EGFP^ mice using Bay 11-7085 (200 µg/kg i.p.; 3 injections per week for 14 days). Interestingly, although we previously showed that Bay 11-7085 attenuated commensal bacteria-induced colitis in IL-10^−/−^ mice, histological analysis showed that *C. jejuni*-induced colitis still developed in mice treated with the NF-κB inhibitor (data not shown).

## Discussion

In this study, we define host responses to *C. jejuni* infection using gnotobiotic technology. Since the transcription factor NF-κB is a key component of host responses to various infectious microorganisms, we focused our attention on the pattern of NF-κB activity using IL-10^−/−^; NF-κB^EGFP^ mice. *C. jejuni* mono-associated IL-10^−/−^ mice developed rapid and severe colitis, even in the presence of low bacterial inoculum (10^2^ cfu/mouse). This finding shows that *C jejuni* itself has the capacity of inducing disease and that the contribution of other intestinal microorganisms is likely not needed. Another important observation is the difference of disease onset between gnotobiotic IL-10^−/−^; NF-κB^EGFP^ mice (14 days, severe bloody inflammation) and mice housed in regular specific pathogen free conditions (>30 days, mild inflammation) [18], [19]. Clearly, these differences relate to the ease of *C. jejuni* to establish a niche in germ free mice whereas the complex intestinal microbiota present in SPF conditions severely restrain *C. jejuni* expansion/colonization.

Although IL-10^−/−^ mice are susceptible to bacterial infection/colonization, it is important to underline the selective nature of their host responses to various bacterial strains. For example, *Helicobacter hepaticus*, a potential pathogenic microorganism that has been reported to induce colitis, hepatitis, and hepatocellular carcinoma in numerous murine models including A/JCr, SCID/NCr and RAG-2^−/−^ mice failed to induce colitis or to potentiate colitis in conventionalized IL-10^−/−^ mice [24]. Also, the gram-negative *Bacteroides vulgatus* bacterium fails to trigger significant inflammation in IL-10^−/−^ mice [21] whereas the same organism triggers severe colitis in HLA-B27/β2 microglobulin transgenic rats [25]. Moreover, we recently showed that in a model of colitis-associated colon cancer, *B. vulgatus* failed to promote tumor development in IL-10^−/−^ mice whereas numerous adenomas are present in conventionalized- IL-10^−/−^ mice [26]. In addition to the differential impact of bacteria on colitis in IL-10^−/−^ mice, the onset of disease is also selectively modulated by colitogenic bacteria. For example, *Enterococcus faecalis* induced a slow-onset (10–12 weeks) of distal colon predominant inflammation, which progressed to severe distal colitis and duodenal inflammation by 24 weeks in IL-10^−/−^ mice [27]. As opposed, *Escherichia coli*-monoassociated IL-10^−/−^ mice developed moderate to severe disease by 16 weeks [27]. In all of the above gnotobiotic experiments with colitogenic bacteria, none of the mice developed rectal bleeding or bloody diarrhea. These findings clearly illustrate the selective nature of IL-10^−/−^ mice host responses to microbial colonization/infection. In essence, not all bacterial strains induce intestinal inflammation in this model and the onset/location of inflammation is often bacterial strain specific. The key feature of our gnotobiotic IL-10^−/−^ model is the rapidity by which *C. jejuni* promotes intestinal inflammation. *C. jejuni* monoassociated IL-10^−/−^ mice developed moderate (5 days) to severe (14 days) intestinal inflammation (cecal, proximal and distal colon) accompanied with bloody diarrhea, an onset never observed with any non-pathogenic bacterial strains utilized in the laboratory so far. These findings indicate that *C. jejuni* responses in IL-10^−/−^ mice are unique to the biology of the microorganism and are not reproduced by other commensal/pathogenic bacteria studied so far in this model (*E. coli*, *E. feacalis*, *H. hepaticus*, *B. vulgatus* or *P. fluorescens*). Consequently, gnotobiotic IL-10^−/−^ mice may represent a novel means to uncover pathways and regulatory host response mechanisms intrinsically associated with *C. jejuni* pathogenesis. Some of the pathways may be clinically relevant but more investigation will be needed to confirm their roles.

Toll-like receptor (TLR) and nucleotide-binding oligomerization domain (Nod) proteins are essential signaling pathways involved in innate/adaptive host responses to various commensal and pathogenic bacteria [28]. For example, TLR4 detects the presence of extracellular bacterial products (LPS), whereas NOD2 senses peptidoglycan and its by-product muramyldipeptide (MDP). In addition, NOD2 senses live intracellular pathogenic microorganisms such as *Salmonella enterica*, *Listeria monocytogenes*, *Mycobacterium tuberculosis* and *Streptococcus pneumoniae* [29]–[32]. This host response is central to the elimination of the damaging agents and to the reestablishment of homeostasis. For example, defective Nod2 signalling impaired *Salmonella typhimurium* clearance by intestinal epithelial cells in vitro and decreased host responses to *Listeria monocytogenes* infection in vivo [30], [31]. Similarly, defective TLR/MyD88 signalling enhanced host susceptibility to *Mycobacterium avium*, *Citrobacter rodentium*, *C. jejuni* infection and *S. typhimurium* clearance [33]–[35]. Interestingly, we observed a strong induction of NOD2 mRNA in *C. jejuni* infected CMT-93 cells suggesting a potential role for this innate sensor in controlling infection. However, preliminary studies showed that similar to WT, NOD2^−/−^ mice housed in SPF conditions failed to develop colitis following *C. jejuni* infection (data not shown). This finding suggests that this innate sensor is not critical for host response to *C. jejuni* and that other signalling pathways may be at play in this model.

An important TLR/NOD down-stream effecter target is the NF-κB transcriptional system, which controls the expression of numerous genes involved in both innate and adaptive responses. We recently demonstrated that TLR/MyD88 signalling to the NF-κB transcriptional system is critical for commensal bacteria-induced colitis in IL-10^−/−^ mice [20], [36]. Interestingly, mice partially deficient for NF-κB activation (p50^−/−^;p65^+/−^) exhibit evidence of *C. jejuni*-induced gastroenteritis whereas wild-type mice remain healthy [14]. These findings together with our results suggest that activation of NF-κB dependent genes may be an important feature of host responses to *C. jejuni* infection. However, because p50^−/−^;p65^+/−^ mice still have a functional p65 allele and are therefore capable of inducing a subset of NF-κB dependent genes, the relationship between this transcription factor and *C. jejuni*-induced disease remains to be defined.

We show that *C. jejuni* induced MIP-2, IL-6, TNF and IL-12p40 gene expression was dependent on activation of NF-κB signalling in murine colonic CMT-93 cell line and BMDC. Moreover, a strong increase in colonic EGFP expression was observed in *C. jejuni*-infected IL-10^−/−^; NF-κB^EGFP^ mice compared to IL-10^wt/wt^; NF-κB^EGFP^ mice. Surprisingly, pharmacological NF-κB inhibition using Bay 11-7085 failed to prevent *C. jejuni*-induced colitis. This is in sharp contrast with the same NF-κB inhibitor attenuating *E. faecalis/E. coli*-induced EGFP expression and development of colitis in IL-10^−/−^ mice [20]. Given the extent of NF-κB activation in our model, one could predict that blocking NF-κB activity would either exacerbate or attenuate *C. jejuni*-induced colitis. One possible explanation for the lack of clear effect is that *C.jejuni*-induced NF-κB activity initiates both protective and deleterious host responses in this model. For example, intestinal-derived NF-κB signalling may be important for the induction of antimicrobial factors and to maintain proper barrier function in response to *C. jejuni* infection. On the other hand, production of NF-κB -dependent inflammatory mediators by lamina propria immune cells may be part of the inflammatory process observed in *C. jejuni*-infected mice. The net effect of NF-κB inhibition on *C. jejuni* pathogenesis is likely to be the sum of both of these positive and negative forces. One would need to use mice with tissue specific deletion of key NF-κB signaling molecules to tease out the contribution of this transcription factor on *C. jejuni*-induced pathogenesis. In summary, using gnotobiotic IL-10^−/−^; NF-κB^EGFP^ mice, we show that *C. jejuni* triggers rapid and severe intestinal inflammation accompanied by bloody diarrhea. Although *C. jejuni* infection leads to activation of NF-κB both in vitro and in vivo, blockade experiments failed to clearly assess the contribution of this transcription factor in *C. jejuni*-induced pathogenesis. Experiments using mice with intestinal-specific deletion of I kappa β kinase β (IKKβ) on the background of IL-10^−/−^ mice are currently underway and should help determine the role of NF-κB on *C. jejuni*-induced pathogenesis.
